# The influence of internal pressure and neuromuscular agents on *C. elegans* biomechanics: an empirical and multi-compartmental *in silico* modelling study

**DOI:** 10.3389/fbioe.2024.1335788

**Published:** 2024-03-15

**Authors:** Clara L. Essmann, Muna Elmi, Christoforos Rekatsinas, Nikolaos Chrysochoidis, Michael Shaw, Vijay Pawar, Mandayam A. Srinivasan, Vasileios Vavourakis

**Affiliations:** ^1^ Department of Bioinformatics and Molecular Genetics, University of Freiburg, Freiburg, Baden-Wuerttemberg, Germany; ^2^ Department of Computer Science, University College London, London, United Kingdom; ^3^ Software and Knowledge Engineering Lab, Athens, Greece; ^4^ Department of Mechanical Engineering and Aeronautics, University of Patras, Patras, Greece; ^5^ National Physical Laboratory, Teddington, United Kingdom; ^6^ Department of Mechanical and Manufacturing Engineering, University of Cyprus, Nicosia, Cyprus; ^7^ Department of Medical Physics and Biomedical Engineering, University College London, London, United Kingdom

**Keywords:** *Caenorhabditis elegans*, finite element method, biomechanics, atomic force microscopy, aldicarb, osmotic shock, optogenetics

## Abstract

The function of a specific tissue and its biomechanics are interdependent, with pathologies or ageing often being intertwined with structural decline. The biomechanics of *Caenorhabditis elegans*, a model organism widely used in pharmacological and ageing research, has been established as biomarker for healthy ageing. However, the properties of the constituent tissues, and their contribution to the overall mechanical characteristics of the organism, remain relatively unknown. In this study we investigated the biomechanics of healthy *C. elegans* cuticle, muscle tissue, and pseudocoelom using a combination of indentation experiments and *in silico* modelling. We performed stiffness measurements using an atomic force microscope. To approximate the nematode’s cylindrical body we used a novel three-compartment nonlinear finite element model, enabling us to analyse of how changes in the elasticity of individual compartments affect the bulk stiffness. We then fine-tuned the parameters of the model to match the simulation force-indentation output to the experimental data. To test the finite element model, we modified distinct compartments experimentally. Our *in silico* results, in agreement with previous studies, suggest that hyperosmotic shock reduces stiffness by decreasing the internal pressure. Unexpectedly, treatment with the neuromuscular agent aldicarb, traditionally associated with muscle contraction, reduced stiffness by decreasing the internal pressure. Furthermore, our finite element model can offer insights into how drugs, mutations, or processes such as ageing target individual tissues.

## 1 Introduction

The nematode *C. elegans* is a powerful model organism for the study of different physiological processes including neuronal signalling, stress response and ageing, all of which ultimately manifest in behavioural changes. Moreover, nematode behaviour has been extensively studied to test or investigate drugs, and to analyse the effects of genetic mutations. Automated, multidimensional *Caenorhabditis elegans* motion trackers by our group and others paired with machine learning have generated databases for behavioural phenotypes and tracked life and health span effects of genetic and pharmacological interventions (Stroustrup et al., 2013; Yemini et al., 2013; Javer et al., 2018; Shaw et al., 2018).

Among these tracked behavioural responses, locomotion plays a major role. The nematode’s locomotion involves a complex interplay between the neuronal network, contracting muscles, and the surface traction between the cuticle and the culture medium. Notably, locomotion also depends on the biomechanics of the tissues. As the animal ages, its body gradually becomes softer and loses the ability to move. We have previously analysed the biomechanics of ageing animals by atomic force microscopy and revealed a correlation between loss of stiffness and diminished mobility, establishing stiffness as a reliable biomarker for healthy ageing (Essmann et al., 2020). Since our stiffness calculations were based on the well-established Hertzian model for contact mechanics (Sneddon, 1965) by assuming the nematode’s body being a single uniform tissue, it resulted in one bulk stiffness indifferent to individual tissue mechanics (Essmann et al., 2020). Ultimately, to differentiate between the decay of individual tissues, we need to map the properties for each tissue layer.

**FIGURE 1:**
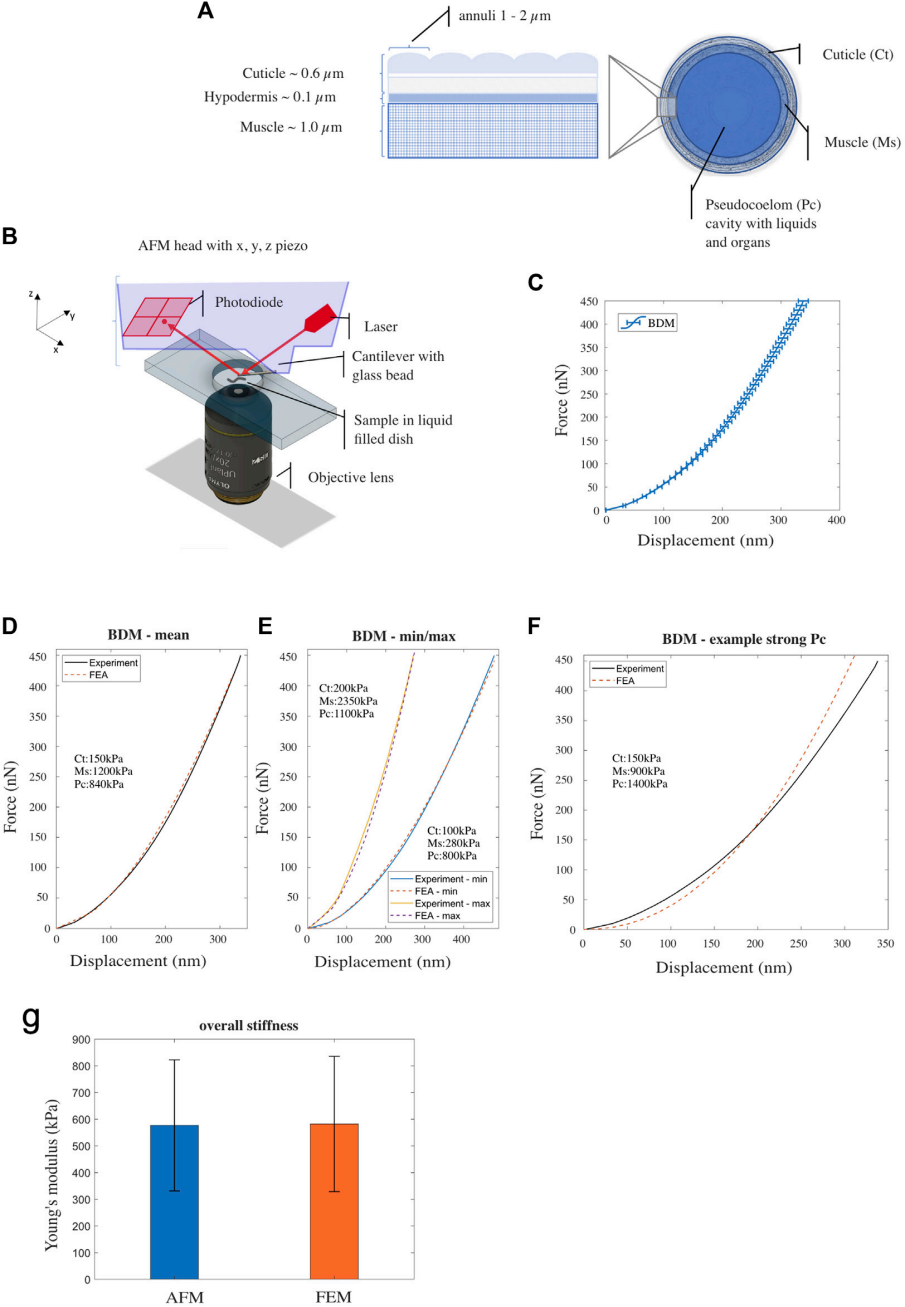
**(A)** Illustration of *Caenorhabditis elegans*’ body composition—structure of the outer layers (cuticle, hypodermis and muscle tissue) (left) and a cross sectional view (right). **(B)** Schematic drawing of the AFM probing device setup. **(C)** Mean experimental F-D curve for *Caenorhabditis elegans*’ treated with BDM, measured using AFM. Horizontal bars denote standard error from the mean [*n* = 44; replicates = 3]. **(D, E)** Experimental (AFM) and simulated (FE model) F-D response curves corresponding to nematodes with mean, the minimum/maximum stiffness respectively. **(F)** Experimental data (BDM control) and simulated F-D response curve from the FE model with decreased Young’s modulus for the muscle component and increased Young’s modulus for the pseudocoelom. **(G)** Overall Young’s modulus of *Caenorhabditis elegans*—comparison between AFM-software model (Hertz-Sneddon) and FE model simulation. Vertical error bars denote standard deviation from the mean.

*Caenorhabditis elegans* is composed of distinct tissue layers, including the cuticle, hypodermis, muscles and the pseudocoelom (Figure 1A). The latter is a fluid filled cavity enclosing internal organs, which contributes to the overall biomechanics and structural support of the nematode through hydrostatic pressure (Altun and Hall, 2009). Each tissue layer is unique in its molecular and cellular composition thus likely different in stiffness, but very little is known about the local material properties and stiffness of these layers, and how they contribute to the overall body stiffness. Knowing the stiffness of each layer is important to examine the impact of specific mutations (e.g., mutations affecting muscle function or the extracellular matrix) external influences (food, temperature, salt content of the media), or processes such as ageing on tissue properties. This lack of knowledge arises from the challenge of directly measuring the deeper layers using current *in vivo* experimentation procedures. Recently, researchers analysed the stiffness of the dissected animal cuticle and how it is affected by collagen mutations or ageing using tensile test techniques (Rahimi et al., 2022). Deeper layers, such as the muscle layer, and hydrostatic pressure, have been experimentally modified to analyse their contribution to the overall stiffness of the animal, albeit their individual stiffness values have not been determined (Park et al., 2007; Petzold et al., 2011; Backholm et al., 2013). Petzold et al. used piezoresistive cantilevers to find that chemically- or optogenetically-induced muscle contractions significantly increased the bulk stiffness, while muscle relaxation reduced it. Moreover, Park et al. described that puncturing the animal with a fine needle to release the hydrostatic pressure had modest effect on the overall biomechanics. Our approach to overcome these limitations is to use computational modelling and to develop a new biomechanical model capable of assigning mechanical properties to distinct individual layers.

The biomechanics of *C. elegans* have been modelled in various ways based on experimental interrogations (Park et al., 2005; Park et al., 2007; Nakajima et al., 2009). These studies incorporated a custom-designed MEMS device and an environmental-scanning electron microscope (E-SEM) to measure *C. elegans* stiffness at the microscopic level. Utilising experimentally measured force-displacement (F-D - indentation depth) data, Park et al. employed a linearized Hertzian contact model, whereas Nakajima et al. employed a Euler-buckling theory model to estimate nematode bulk tissue stiffness. Further, Gilpin et al. (2015) employed a linear-spring mechanical analogue to explicitly model hydrostatic pressure, by taking the difference between the external and the desired internal pressure, to explore *C. elegans* whole-body biomechanics. Our previous study (Elmi et al., 2017) introduced a three-dimensional finite element (FE) model, assigning distinct stiffness values to an outer (cuticle, hypodermis, muscle) and an inner layer (pseudocoelom). This model was based on linear-elastic isotropic, homogeneous soft matter. More recently, Sanzeni et al. (2019) modelled *C. elegans*’ body as a tapering cylinder that consists of an outer shell structure (i.e., the cuticle, hypodermis, and body wall muscles) and an inner (tube) structure of the pseudocoelom with the intestine and gonad gland. To simplify their structural model, they employed a Hookean stress/strain constitutive law for the solid body of the animal. A more advanced model that uses a second-order elasticity theory to capture larger amplitude deformations and material nonlinearity was proposed by Du et al. (2023).

Here, we combine a new experimental procedure based on atomic force microscopy with the development of a new multi-compartmental FE model. We demonstrate the importance of internal pressure as the primary determinant of overall body stiffness and that hyperosmotic shock significantly reduces body stiffness. Moreover, our data show that the neuromuscular agents aldicarb and tetramisole reduce the internal pressure. This has important implications for our understanding of the effects of neuromuscular agents and their effect on the biomechanics of *C. elegans*. Moreover, the model can be used to predict how mutations or pharmacological interventions impact the age-related decline of individual tissues, especially those known to increase lifespan.

## 2 Materials and methods

### 2.1 *Caenorhabditis elegans* laboratory maintenance

*Caenorhabditis elegans* wild-type (N2) and ZX299 animals were obtained from the *Caenorhabditis* Genetics Centre. ZX299 are genetic mutants lin-15B&lin-15A (n765) X with the extrachromosomal expression construct zxEx22 [myo-3p::ChR2(H134R)::YFP + lin-15 (+)] expressing channel rhodopsin in the body wall muscles. All animals were maintained at 20*°*C on NGM plates and fed with OP50 (Brenner, 1974; Stiernagle, 2006). For experimental purposes L4 animals were grown at 20*°*C until reaching the adult stage.

### 2.2 Drugs and osmotic shock treatments

Prior to the force-displacement measurements the animals were treated for 60 min with 15 mg/mL (148 mM) BDM (2,3-butanedione monoxime, Sigma-Aldrich), or 1 mM aldicarb (Sigma-Aldrich), or for 90–120 min with 100 *μ*M (−)-tetramisole hydrochloride (Levamisole, Sigma-Aldrich) in M9 buffer solution until they were no longer moving. For acute hyperosmotic shock treatment, animals were incubated in a 500 mM NaCl solution (NaCl in distilled water) for 1 hour before force measurement containing 15 mg/mL BDM to prevent movement during measurements. For comparison between drugs administered in solution (as described above) *versus* NGM plate, animals were treated with 15 mg/mL BDM in solution or on a NGM plate, or 1 mM aldicarb in solution or on a NGM plate for 60 min. For morphology analysis, animals were treated with either the drug solution or hyperosmotic solution (500 mM NaCl) at the same concentrations as described above. For optogenetic experiments: ZX299 transgenic animals were cultivated on agar plates with OP50 bacteria in the presence of all-trans retinal (Sigma-Aldrich) (Nagel et al., 2005). Animals were treated as described above prior to the force-measurements. For stimulation of channelrhodopsin, animals were illuminated with 450–490 nm light.

### 2.3 Force measurements

#### 2.3.1 Atomic force microscopy (AFM)

Treated animals were transferred to a 1 mm thick 4% agarose bed in a Petri dish and Dermabond glue (2-octyl cyanoacrylate, Suturonline.com) applied carefully to head and tail using the tip of a pulled glass needle as described previously (Essmann et al., 2017). Force measurements were taken from the neck and hip region of the animal, avoiding the mid body or vulva region, see (Essmann et al., 2020). Preparation of the animals and AFM measurements were all performed at RT. Individual force-displacement curves were acquired using a NanoWizard3 AFM (JPK) in force spectroscopy mode (set force 450 nN; 0.5 mm/s indentation speed). All data were captured using a 10 *μ*m diameter glass bead attached to a tipless cantilever of k = 5.79–10.81 N/m stiffness (NSC12 7.5 N/m *μ*Masch produced by sQUBE) to prevent the cuticle from being pierced at larger indentations. Cantilever sensitivity and spring constant were calibrated using the JPK calibration tool (thermal noise method; see the work of Butt and Jaschke (1995)) prior to each experiment.

#### 2.3.2 Micro-force displacement system (μFDS)

For larger amplitude force-displacement experiments we used an in-house customized micromanipulation setup to allow uniaxial indentation of *C. elegans* with a microforce sensing probe (Elmi et al., 2017). Treated animals were mounted on a 2% agarose pad on top of a microscope slide (Figure 3B). To prevent motion during indentation, animals were glued (Dermabond glue, Suturonline.com) on the side to the edge of a coverslip fixed on top of the agarose pad before immersion in M9 buffer. The sample imaged using an upright widefield fluorescence microscope (BX51WI, Olympus) with 20x/1.0 water immersion objective lens (LUMPlanFL N, Olympus) fluorescence filter cube (Semrock) and a sCMOS camera (Orca-Flash4.0 v2, Hamamatsu Photonics). The body of each animal was indented using a microforce sensing probe (FT-S100, FemtoTools) fitted with a tungsten tip. The position of the probe was controlled using a motorized 4-axis stage system (ECS series, Attocube), which allowed precise positioning of the tip within (x, z) and perpendicular (y) to the focal plane of the microscope, as well as adjustment of the in-plane tilt. Animals were mounted on a separate kinematic stage system decoupled from the microscope body and the probe.

#### 2.3.3 AFM and μFDS data analysis

Raw AFM data were analysed using JPK analysis software. All individual force curves were processed to zero the baseline, to determine tip-sample contact point and to subtract displacement of the tip due to cantilever bending. To calculate the Young’s Modulus, the mean, minimum and maximum force-indentation curve (Figure 1D) was further analysed by fitting the Hertz/Sneddon model for contact mechanics to the entire curve using the JPK software and by taking the indenter shape (10 *μ*m diameter bead) into account (see Figure 1F). The μFDS directly recorded the force data as displayed in the graphs with minimal processing: Data series were truncated to exclude force and displacement readings captured during approach of the probe to the cuticle and readings zeroed just before the probe tip made contact with the animal (confirmed optically).

To calculate the nematode bulk stiffness from either the AFM and the μFDS force-displacement data, linear regression was applied on the data within the [0.5 *δ*_max_, *δ*_max_] range, where *δ*_max_ the maximum displacement value from the AFM and μFDS data.

#### 2.3.4 Imaging and analysis

Body measurements: Images were acquired using a CMOS camera (Orca-Flash4.0 v2 Hamamatsu Photonics). The ImageJ plugin WormSizer was used for detecting the animal size, measuring body length and diameter, and body volume (Jung et al., 2012; Moore et al., 2013).

### 2.4 *Caenorhabditis elegans* FE model

To simulate nematode biomechanics, an advanced *C. elegans* finite element (FE) model was developed using the proprietary software ABAQUS/Standard (SIMULIA, 2014). The proposed 3D FE model overcomes two major simplifications of published biomechanical models: (*a*) it distinguishes the major tissue components of *C. elegans* that primarily contribute to the body biomechanics, which are modelled individually (with separate model parameters). (*b*) Our FE model accommodates nonlinear (material model) biomechanical behaviour and the nonlinearities that appear from the mechanical interactions with components of the testing device (i.e., the AFM tip). Part of the nematode body was approximated in ABAQUS as an idealized cylinder (see Supplementary Text S1) subdivided into three distinct tissue compartments corresponding to the cuticle and dermis, the muscle tissue, and the pseudocoelom. Each tissue compartment was modelled using separate material properties (i.e., stiffness) based on a Green-elastic, neo-Hookean constitutive model (see Supplementary Text S1). Next, for the FE discretization of the *C. elegans* model, a hexa-dominant 3D mesh was generated in ABAQUS, with a finer mesh used close to the region where the tip contacts the cuticle (see Supplementary Figure S5 in Supplementary Text S1). A mesh sensitivity and convergence analysis found the optimal FE mesh had a 0.4 *μ*m minimum edge size (see Supplementary Figure S6 in Supplementary Text S1). Finally, proper contact and boundary conditions were defined to reduce the size of the computational domain and replicate the constraints applied to the nematode movement *in vivo* during testing (see Supplementary Text S1). Computer simulations were run on a Dell workstation with an Intel(R) Core(TM) i7-6800K CPU (3.40 GHz) and 64 GB of RAM.

## 3 Results

### 3.1 Three-compartment nonlinear biomechanical FE model of *Caenorhabditis elegans*

We modelled *C. elegans* using the FE method as an idealized cylindrical structure, which consists of four distinct concentric compartments; the cuticle (width 0.6 *μ*m) is the outermost layer that serves as an exoskeleton, the hypodermis (width 0.1 *μ*m) a very thin cellular layer, the muscle tissue (width 1.0 *μ*m), and the pseudocoelom (diameter 26.3 *μ*m), a fluid filled cavity with internal organs and gonads (Lints and Hall, 2009; Wolkow and Hall, 2013; Altun and Hall, 2023), see Figure 1A. Previously we used a simple two layered model and large force-displacement (F-D) measures of up to 14 *μ*m to describe *C. elegans* biomechanics (Elmi et al., 2017); thus, combining the three outer layers into one compartment. Here, we wanted to elucidate on the role of the distinct components of this previously named ‘outer’ layer and their contribution to the overall stiffness. We therefore required a force sensing system in the range of nanonewtons that is capable of indentations in the range of nanometres to a few micrometres. For this purpose, we used an atomic force microscope (AFM; Figure 1B), to produce data to inform and validate the FE model (see Supplementary Text S1).

As in our previous studies (Elmi et al., 2017; Essmann et al., 2017), we used 2,3-butanedione monoxime (BDM) to immobilise animals during AFM analysis. BDM is a chemical that has been described to relax muscles (Petzold et al., 2011; Backholm et al., 2013). Using a 10 *μ*m bead attached to a tipless cantilever to avoid damaging or penetrating the cuticle during the indentation process, we applied a set force of 450 nN and recorded individual force-indentation curves. The mean indentation depth at this force was 338 nm ± 8.4 SEM (*n* = 44; Figure 1C). Subsequently we employed our FE model to simulate the AFM indentation test and to reproduce the F-D plots obtained from the experiments. Following an Edisonian approach (Dandekar et al., 2003; Wills, 2019), we attempted through the simulations to interrogate and estimate the model parameter values (Young’s modulus) for each of the three tissue compartments of the FE model: the cuticle (cuticle and hypodermis), the muscle, and the pseudocoelom. Details however regarding the computational procedure to estimate the parameter values are provided in the Supplementary Text S1. Figure 1D shows the mean force (nN) to displacement (nm) curve of the experiments (black solid line) compared against the FE-simulated curve (red-dashed line). After optimising for the Young’s modulus of each of the three compartments of the *C. elegans* FE model, we converged to the following set of values that gave the best agreement to the experimental data: 150, 1,200 and 840 kPa for the cuticle, muscle and pseudocoelom respectively (Figure 1D and Supplementary Figure S1A). To explore the range of natural variation between animals’ biomechanics, we attempted to reproduce the two extreme F-D curves of the experiments (min, max) with the FE model, and quantified the difference in Young’s modulus for each tissue compartment (Figure 1E). Taking the natural variation into consideration, the mechanical properties we propose for BDM-treated wild type animals range from 100 to 200 kPa for the cuticle, from 280 to 2,350 kPa for the muscle layer and from 800 to 1,100 kPa for the pseudocoelom (mean values: 150 kPa for the cuticle, 1,200 kPa for the muscle, 840 kPa for the pseudocoelom).

Figure 1F shows a simulated F-D curve generated from the FE model with Young’s modulus values for the muscle tissue set to 900 kPa and for the pseudocoelom set to 1,400 kPa. The nematode’s bulk stiffness predicted by the FE model is lower at the indentation range < 150 nm and increases rapidly at indentation > 200 nm, with poor agreement with the experimental F-D curve (solid line in Figure 1F). To validate our FE model, we compared the numerically calculated overall Young’s modulus of *C. elegans* to that obtained from the Hertz/Sneddon model using the AFM-data analysis software. We found no significant difference (582 kPa ± 253 kPa *versus* 577 kPa ± 245 kPa respectively) between the AFM measurements and FE model predictions, as shown in the bar plot of Figure 1G. For this, the overall stiffness values of the mean, min and max F-D curves were averaged (see Figure 1F).

### 3.2 Hydrostatic pressure contributes significantly to *Caenorhabditis elegans*’ bulk stiffness

**FIGURE 2:**
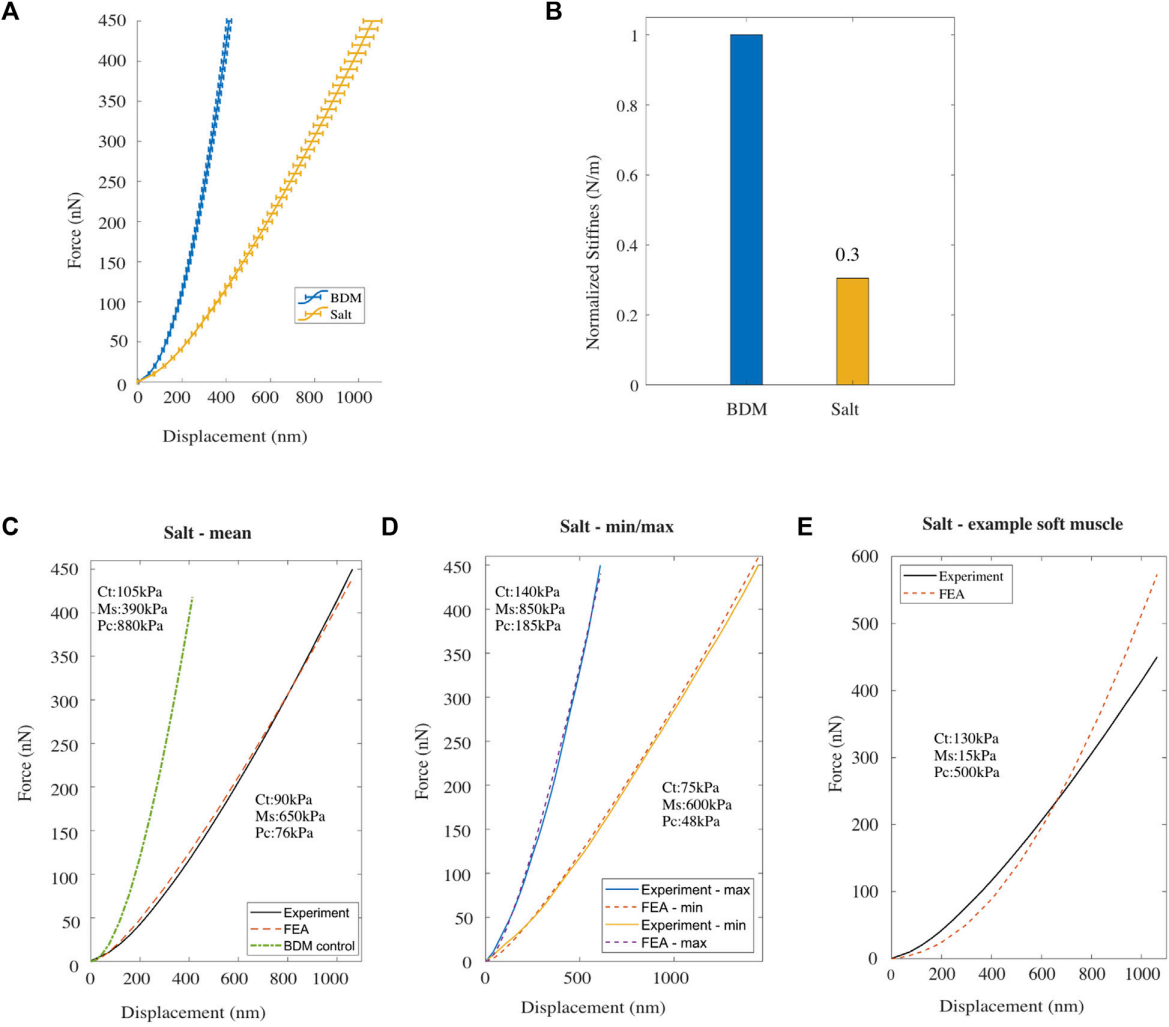
**(A)** Mean F-D curve for *Caenorhabditis elegans* treated with BDM alone (*blue*) [*n* = 48] and treated with BDM and exposed to high salt buffer (yellow) [*n* = 48; replicates = 3], measured using AFM. Horizontal bars denote standard error from the mean. **(B)** Normalized bulk stiffness estimated from the F-D data. **(C, D)** Mean, and the minimum/maximum experimental (AFM) and simulated (FE model) F-D response curves for worms treated with high salt. **(E)** Experimental F-D curve for high salt treated worms and the F-D curve predicted by the FE model with a reduced value for Young’s modulus of the muscle component compared to the BDM treated control.

To elucidate the contribution of internal hydrostatic pressure to the stiffness, we exposed animals to a high salt solution (0.5 M NaCl) to induce hyperosmotic osmotic shock and captured F-D curves using the AFM as previously. In high salt solution the set-point force of 450 nN was reached for a mean indentation depth of 1,063 nm ± 42 SEM (*n* = 46). Under control conditions the same compressive force was reached at an indentation depth of 413 nm ± 11 SEM (*n* = 48), meaning that the animals were significantly less stiff under hyperosmotic shock (Figure 2A). To quantify the difference in bulk stiffness between these two conditions, we employed linear regression on the data for moderate to high indentation depths, corresponding to probe displacement > 200 nm for the BDM tests and > 500 nm for high salt tests, and estimated the bulk stiffness of the nematode. The normalized values are seen in Figure 2B (for absolute values see Supplementary Table S1).

The Young’s modulus for each of the three compartments of the *C. elegans* FE model was then modified to fit the mean experimental F-D curve for high salt-treated animals (Figure 2C). After trial-and-error optimisation, the best fit model parameters were: 90 kPa for the cuticle compartment, 650 kPa for the muscle compartment, and 76 kPa for the pseudocoelom. Comparing these values to the best fit parameters in to the control case (105, 390 and 880 kPa; see green dash-dot line in Figure 2C and Supplementary Figure S1B) we observe a 14% and a 91% drop in the Young’s modulus of the cuticle tissue and the pseudocoelom respectively, whereas the muscle sees an ≈ 67% increase of the Young’s modulus. In addition to the mean F-D curve on Figure 2C, we used the FE model to reproduce the two extreme F-D curves of the experiment (min, max), and again optimised the model to estimate Young’s Modulus for each tissue compartment. The simulations for the high salt-treated wild type *C. elegans* gave Young’s moduli that range within 75–140 kPa for the cuticle, 600–850 kPa for the muscle, and 48–185 kPa for the pseudocoelom.

We then lowered the Young’s modulus for the muscle tissue and increased Young’s modulus for the pseudocoelom and re-ran the FE model. As shown in Figure 2E, the agreement of the numerically predicted F-D curve to the experimental data recorded under high salt was poor. This suggests that the hyperosmotic shock caused by salt had the largest impact on the pseudocoelom by reducing its pressure, attributed to loss of water due to osmosis, resulting in reduced volumetric resistance in that compartment. In addition, high salt treatment also increased muscle stiffness in line with previously reported results (Park et al., 2007). In conclusion, the overall body stiffness is significantly reduced in response to salt exposure.

### 3.3 Aldicarb treatment reduces *Caenorhabditis elegans*’ bulk stiffness

**FIGURE 3:**
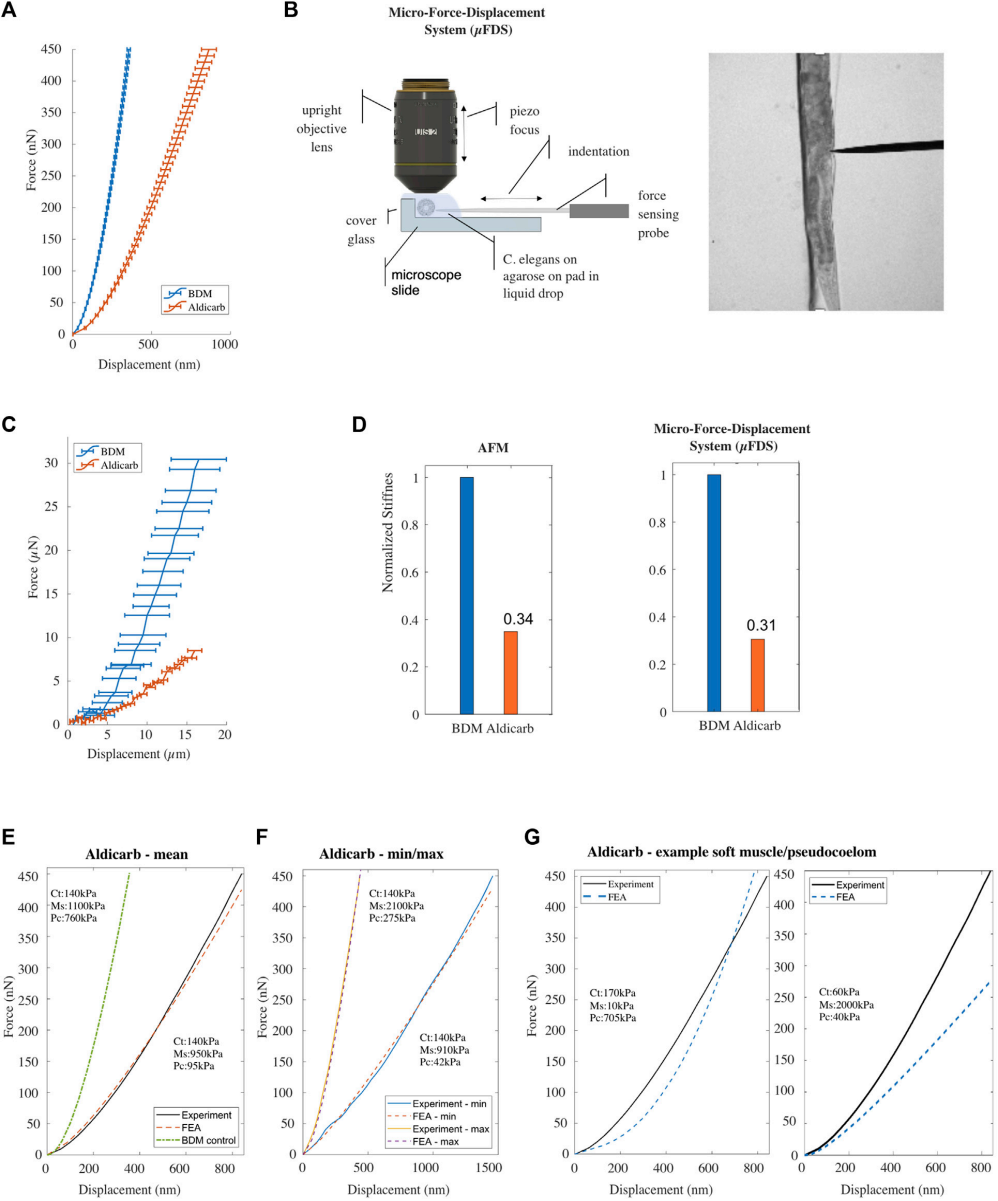
**(A)** Mean F-D data for *Caenorhabditis elegans* treated with BDM [*n* = 68] (*blue*), or aldicarb [*n* = 76; replicates = 3] (*orange*) measured using AFM. Horizontal bars denote error deviation from the mean. **(B)** Schematic drawing of the micro-Force-Displacement System (μFDS) and representative brightfield micrograph showing a worm under indentation (right). **(C)** Mean F-D curves for *Caenorhabditis elegans* treated with BDM [*n* = 5] (*blue*) and aldicarb [*n* = 12] (*orange*) measured using μFDS [replicates = 2]. Vertical bars denote standard error from the mean. **(D)** Normalized bulk stiffness estimated from the F-D curves measured using AFM (left) and μFDS (right). **(E, F)** Mean and minimum/maximum experimental (AFM) and simulated (FE model) F-D curves for worms treated with aldicarb. **(G)** Experimental F-D curve for aldicarb treated worms and the F-D curve predicted by the FE model with a reduced value for Young’s modulus of the muscle component (left) and the pseudocoelom compared to the BDM treated control.

To understand the contribution of the body wall muscle layer to body stiffness we next experimentally modified the muscle tone using the well-known chemical called aldicarb. Aldicarb has been described previously to hyper-contract muscles by blocking the degradation of acetylcholine (ACh) at the neuro-muscular junction of *C. elegans* (Lackner et al., 1999; Nurrish et al., 1999). We treated animals with either aldicarb or BDM as control condition until immobilized and measured their stiffness using the AFM (Figure 3A).

The mean F-D curve of aldicarb-treated animals showed a maximum indentation depth of 864 ± 47 nm (*n* = 76) for a total compressive force of 450 nN, compared to 356 nm ± 11 nm (*n* = 68) for the BDM-treated animals. Contrary to our expectations, this result suggests that aldicarb-treated animals, although assumingly hyper-contracted, are softer than BDM-treated animals (Figure 3A). We hypothesized that when an animal shrinks due to muscle hypercontraction, the cuticle of the animal folds, thereby, allowing for a margin of the corresponding tissue to start to be indented without significant compressive force being applied and therefore making the animal appear softer than in its uncontracted state. Based on this explanation, the stiffness should increase for larger indentations. To test this, we employed a Micro-F-D System (μFDS) as a second force-indentation tool (Figure 3B) able to indent animals up to 20 *μ*m (Elmi et al., 2017). BDM-treated animals were mounted on top of an agarose pad against the edge of a glass coverslip to provide a rigid surface when indenting the animal from the side with a uniaxial force sensor (Figure 3B). Using μFDS, we measured F-D curves for indentations of up to 16 *μ*m. At an indentation depth of 16 *μ*m, we measured a mean compressive force of 29.3 *μ*N ± 3.5 SEM (*n* = 5) for *C. elegans* treated with BDM, whereas for when treated with aldicarb the same indentation resulted in a compressive force of only 8.5 *μ*N ± 0.9 SEM (*n* = 12; Figure 3C). Hence even at larger indentations, aldicarb treated animals are softer than BDM-treated animals, and cuticle folding due to muscle hyper-contraction does not seem to explain the effect of aldicarb on stiffness. To quantify the difference in bulk stiffness between these two treatment conditions, and to compare data from AFM and μFDS setups, we employed linear regression at moderate to high indentation depths to estimate the bulk stiffness using F-D data for indentations > 10 *μ*m for the μFDS data set, and > 200 nm for the BDM and > 300 nm for the aldicarb AFM data set. The normalized values (Figure 3D; for absolute values see Supplementary Table S1) indicate that the bulk stiffness of aldicarb-treated animals is decreased by 66% when compared to BDM-treated animals. To further investigate the effect of the neuromuscular agent, untreated animals were placed in the μFDS, and F-D data was captured over time after adding aldicarb. The results indicate a gradual decline in stiffness to reaching 40% of the initial value 150 min after treatment (Supplementary Figure S5).

This unexpected result prompted us to investigate the effect of tetramisole on animal stiffness, another chemical known to activate cholinergic receptors on the body wall muscles causing muscle hyper-contraction (Lewis et al., 1980). Similarly to aldicarb-treated animals, data captured using AFM and μFDS systems for tetramisole-treated animals show that they are significantly softer than BDM treated animals (see Supplementary Figure S2).

We modified the Young’s modulus for all three compartments of the FE model to reproduce the F-D curve for aldicarb-treated animals (Figure 3E). The numerically estimated Young’s modulus for the cuticle (140 kPa) and the muscle (950 kPa) compartments varied by less than 10% from the corresponding values for BDM-treated animals (Supplementary Figure S1C). However, the estimated Young’s modulus for the pseudocoelom was 94 kPa, and dramatically lower than the 760 kPa for the pseudocoelom compartment of the BDM-treated animals. To also explore the range of natural variation between the aldicarb-treated animals, we re-optimised the model to fit the two extreme experimental (min, max) F-D curves. The natural variation for the pseudocoelom was 42 kPa–275 kPa, for the muscle tissue 910–2,100 kPa, whilst the Young’s modulus of the cuticle remained close to 140 kPa (Figure 3F). We also attempted to fit the experimental data by softening the muscle tissue and stiffening the pseudocoelom, or inversely by stiffening the muscle tissue and softening the pseudocoelom. As seen in Figure 3G the simulated F-D curves (blue dotted lines) in both cases compared poorly to the mean experimental F-D curve for the aldicarb-treated animals (black solid line).

To conclude, rather than increasing stiffness, treating *C. elegans* with aldicarb or tetramisole softened the animal. Fitting the experimental data using our multi-compartment FE model indicates that aldicarb significantly reduced the stiffness of the pseudocoelom without significantly changing the muscle layer compared to the control condition. This suggests that aldicarb has additional effects beyond inducing muscle contraction.

### 3.4 Osmotic shock and aldicarb treatment reduce *Caenorhabditis elegans* size

**FIGURE 4:**
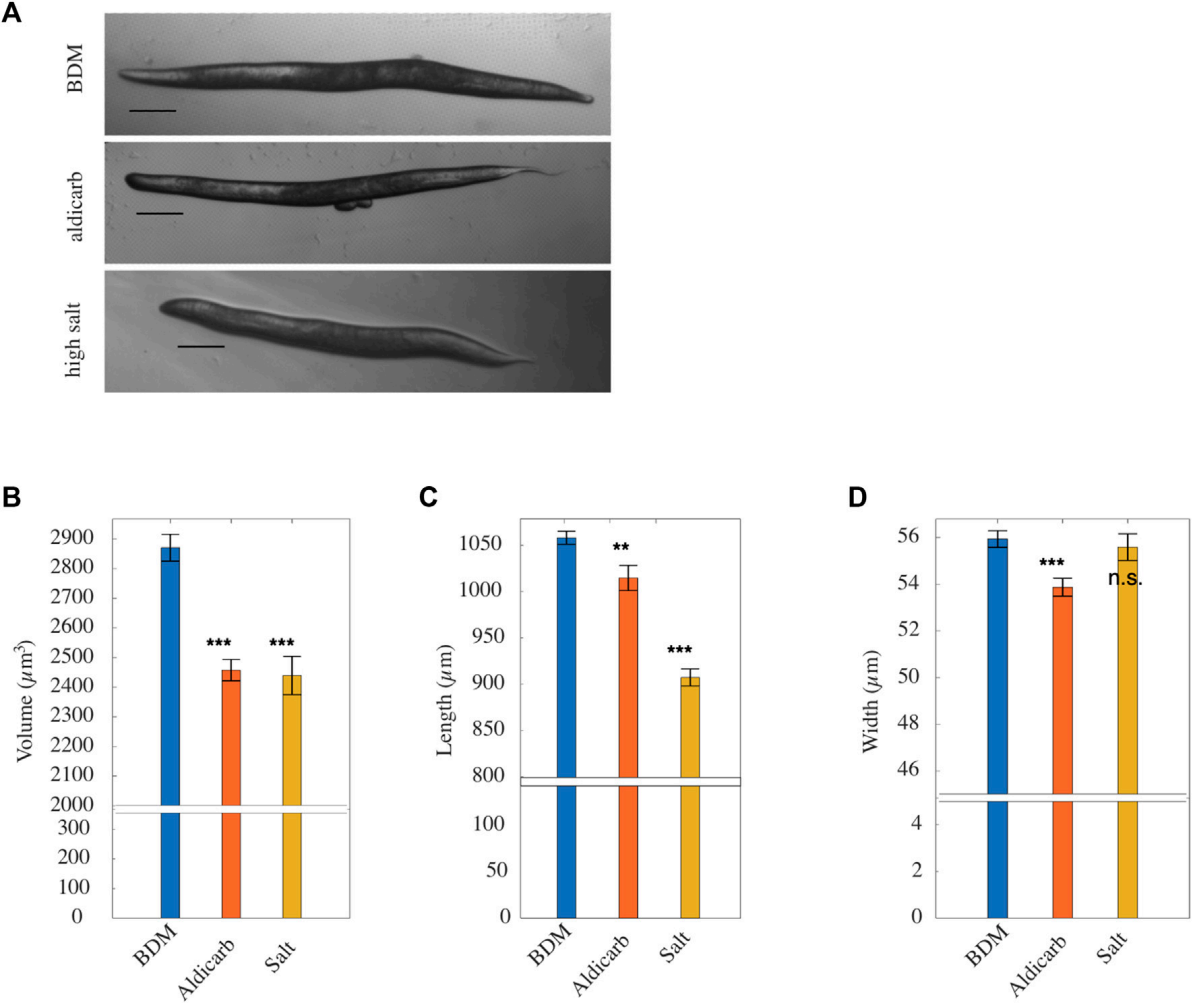
**(A)** Representative brightfield micrographs, ordered from top to bottom respectively, of worms paralysed in BDM, treated with aldicarb and high salt (scale bar: 100 *μ*m). **(B)** Mean volume, **(C)** mean length, **(D)** mean width of worms paralyzed with BDM (*blue*), aldicarb (*orange*) or high-salt (*yellow*) solution [*n* = 64, 49 and 24 respectively; replicates = 3]. Vertical bars denote standard error from the mean. *p* values are indicated as follows: n.s. = not significant, **≤0.01–0.001, and ***≤0.001. Results were determined by two-tailed *t*-test.

Our FEM simulations indicated that animals treated with aldicarb loose most of their pseudocoelom stiffness. The reduced stiffness closely resembled that of animals exposed to an osmotic shock (Figure 2A) which causes loss of water and subsequently shrinkage. (Park et al., 2007). To understand more about the additional effects of aldicarb, we measured changes to the length, width and volume of animals exposed to aldicarb or osmotic shock compared to BDM-treated controls (Figure 4A). Both osmotic shock and aldicarb treatment significantly decreased animal size with measured volumes of 2,457.34 mm^3^ ± 35.9 SEM and 2,439.35 mm^3^ ± 64.6 SEM respectively, compared to 2,870.41 mm^3^ ± 45.1 SEM for animals treated with BDM alone (*n* = 24, 49 and 67 respectively; Figure 4B). In contrast to animals subject to osmotic shock, aldicarb-treated animals reduced more in width (53.87 ± 0.39 *μ*m, compared to 55.59 ± 0.57 *μ*m) than length (1,014.76 ± 13.57 *μ*m compared to 907.22 ± 9.22 *μ*m; Figures 4C, D). It has been reported previously that animals exposed to aldicarb on plates shrink over time (Opperman and Chang, 1991; Miller et al., 1996; Lackner et al., 1999; Nurrish et al., 1999; Glenn et al., 2004; Mahoney et al., 2006). When we compared animals paralysed on aldicarb-containing plates *versus* aldicarb solution we found that animals exposed to aldicarb on plates shrank more with a reduced length and volume (Supplementary Figure S3A). Based on analysis of AFM F-D curves these animals also tended to be 13% softer than animals paralysed in aldicarb solution (Supplementary Figures S3B, C). We observed no difference in stiffness or volume between animals treated with BDM in solution *versus* plate, although we found an ≈ 10% difference in length (Supplementary Figures S3D, F).

### 3.5 Optogenetically controlled muscle contraction increases *Caenorhabditis elegans* bulk stiffness

**FIGURE 5:**
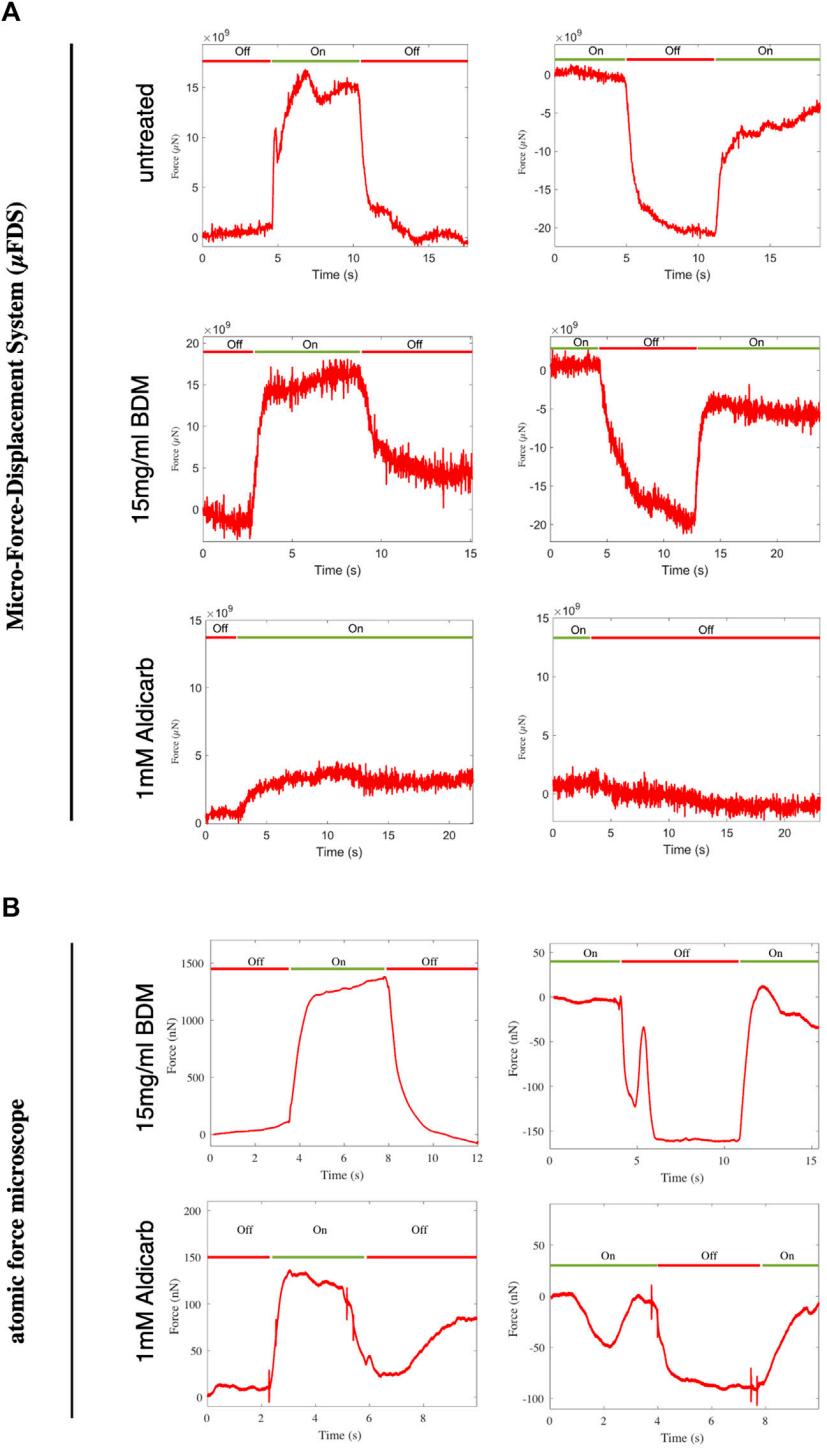
Force measured at a fixed indentation for indentation-clamped transgenic worms expressing ChR2 in muscle tissue. **(A)** μFDS measurements for untreated, BDM or aldicarb treated worms (light off: Off (*red*); light on: On (*green*)). **(B)** AFM measurements for BDM or aldicarb treated worms.

Our data show that, consistent with previous reports (Mulcahy et al., 2013; Izquierdo et al., 2021), when aldicarb is administered in solution or on a plate the animals shrink. In contrary to expectations, we measured a decrease in animal stiffness following aldicarb exposure. Our FEM simulations suggest the decrease in stiffness arises due to a change in the properties of the pseudocoelom (Figure 3E). It is possible that muscle contraction is not measurable with our force-indentation set-ups, or that chemically induced muscle contraction is accompanied by other effects and, in combination, this does not result in tissue stiffening. To investigate this possibility, we employed optogenetics as an alternative, non-chemical, tool to induce muscle contraction, using a strain expressing a channelrhodopsin-2 (ChR2) variant in its muscle tissue (Schultheis et al., 2011). ChR2 is an ion channel that responds to blue light by opening and allowing ions to enter the cell. When expressed in muscle cells ChR2 enables control of muscle contraction (AzimiHashemi et al., 2014). To test whether we could detect stiffness change associated with muscle contraction, we analysed untreated animals expressing channel rhodopsin using the μFDS setup. The AFM system was unsuitable for such measurements due to the uncontrolled, large movements of non-treated animals. We used the force sensor to indent and clamp the animal. After a few seconds of baseline recordings, the animal was illuminated for 7–8 s with blue light to induce muscle contraction (Figure 5A). Illumination (On) resulted in a sharp rise in the compressive force sensed by the force sensing tip. When the blue light was turned off (Off) the force reading declined sharply. The same trend was observed when the force reading was set to zero with blue light switched on: switching it off led to a decrease in measured force, switching it back on to an increase in force (On-Off-On). These results indicate that the μFDS set-up is sufficiently sensitive to measure changes in muscle tone and stiffening following ChR2 mediated muscle contraction (see Supplementary Figure S4.

Next, we investigated whether animals treated with aldicarb, or BDM retained the ability to contract their muscles when under ChR2-induced muscle activation. Assuming BDM immobilises animals by relaxing the muscles, channel rhodopsin activation should still lead to muscle contraction and hence stiffening of the animal. On the contrary, aldicarb-treated animals (with hypercontracted muscles) would likely not increase their stiffness following ChR2 activation. Using both the μFDS (Figure 5A) and AFM (Figure 5B) set-up, BDM-treated animals showed a robust increase in stiffness upon ChR2 activation in both activation–deactivation sequences (On–Off–On or Off–On–Off). Untreated transgenic animals responded similarly to blue light activation (ure 5A). Hence it is possible to induce and to measure muscle contraction in animals treated with BDM, suggesting BDM leaves muscles in a relaxed or responsive state. Using the μFDS set-up we did not detect a significant increase in stiffness in aldicarb-treated animals following illumination (Figure 5A and Supplementary Figure S4A). Using the AFM set-up, however, we observed an increase in measured compressive force for approximately half of the animals following blue light exposure (Figure 5B and Supplementary Figure S4B). This suggests that responsive aldicarb-treated animals had contractile muscles which were not hypercontracted. For those animals that did not respond, muscles were either hyper-contracted and unable to contract further or were unresponsive due to additional effects of aldicarb. We suggest the latter as the most likely explanation, as ChR2 activation-induced muscle contraction results in a stiffening of the animals, and aldicarb-treated are softer compared to BDM-treated animals (Figure 3).

## 4 Discussion

### 4.1 FE model

In this study we generated empirical F-D data of small-scale indentations in *C. elegans* of up to 1 *μ*m, which we used subsequently to build a *in silico* model to simulate the biomechanics of the animal. In distinction to previously published work, our model can permit to assigning Young’s moduli to three distinct compartments (cuticle, muscle and pseudocoelom) based on the shape of experimentally acquired F-D curves. AFM data indicated that the F-D relation is highly non-linear (see, for example, Figure 1C); hence, we applied pertinent methodological considerations when building our *in silico* model. Our model differs from that of previous works, such as the “homogenous cylindrical shell with internal hydrostatic pressure” model by Park et al. (2007) and the “pressurized cylinder composed of two layers” model by Elmi et al. (2017), both of which assumed *C. elegans* tissues are linear elastic. Our model effectively encompasses the nonlinearities stemming from the contact between the indenting device and the animal’s cuticle, and the inherent nonlinear biomechanical properties of *C. elegans*’ constituent tissues.

Using experimental data to inform and tune the parameters of our *in silico* model, we successfully reproduced experimental F-D curves by varying the Young’s moduli of the different compartments. We were then able to evaluate the contribution of different compartments, and the effect of neuromuscular agents and high salt on tissue biomechanics and *C. elegans*’ bulk stiffness. However, our *in silico* model comes with simplifications with respect to (*a*) the idealized geometrical representation of the nematode’s body, (*b*) the anisotropic biomechanical behaviour of the cuticle and muscle tissue, (*c*) the transport of biofluids in the pseudocoelom, and (*d*) the swelling of tissues due to biochemical factors. To increase the resolution and complexity of the model, new data from experimental modalities would be required which were not available during this work. This could include tensile testing of *ex vivo* specimen cut-outs of the cuticle, or muscle tissue at different orientations to measure anisotropy, and to use optical tomography to measure fluid flow in the gonad and intestinal cavity.

### 4.2 Hydrostatic pressure

Our *in silico* modelling data suggest that the pseudocoelom has a substantial impact on the overall biomechanics of *C. elegans*. Reduction of internal pressure by high salt exposure reduced the Young’s modulus of the pseudocoelom by 91% according to our model. This observation contrasts with the findings of Park et al. (2007) in two ways. Firstly, their study proposed that the mechanics of the ‘outer shell’ was the major contributor to animal stiffness, and secondly, that hyperosmotic stress increased stiffness. How can a deeper compartment like the pseudocoelom affect stiffness? The contribution of an underlying tissue compartment even if not directly indented, in our case the indentation depth reached by the AFM was maximum 1 *μ*m, is relevant to due to the model’s assumed incompressibility of all three compartments. As a result, the deformation of the outer compartment (cuticle) is transferred rigidly to the compartments below. To demonstrate the influence of the pseudocoelom on the F-D curve we decreased the Young’s modulus of the muscle compartment instead of the pseudocoelom. The simulated F-D curve no longer matched the experimental results, being shallower for smaller displacements due to the soft muscle compartment, and steeper for larger displacements due to the stiff pseudocoelom compartment. Secondly, Park and others reported that hyperosmotic shock increased body stiffness due to salt-induced muscle contraction, meaning the reduced internal pressure had lesser impact on the overall body stiffness. In our experiments, exposure to a high salt concentration led to reduced stiffness. A key difference between the two studies is that our animals were treated in solution not on plates, and, in addition, with BDM for complete immobilization during our AFM measurements. BDM, acting as a muscle relaxant, may have interfered with muscle contraction promoted by hyperosmotic conditions. It is worth noting; however, that the body wall muscles of BDM-treated animals remained responsive to light-induced ChR2 in the presence of BDM (Figure 5). However, it is possible that the pathway leading to hyperosmotic-induced muscle contraction acts upstream of BDM, and consequently, our results primarily reflected the effect of a reduced hydrostatic pressure. Nevertheless, our findings are consistent with the observation that a decrease in internal pressure due to cuticle puncture similarly reduces body stiffness (Park et al., 2007).

### 4.3 Aldicarb on stiffness and morphology

The neuromuscular agent aldicarb has been an invaluable tool in *C. elegans* research to investigate neuromuscular function and behaviour. By assessing mobility and paralysis in response to this agent, researchers have gained insights into neuromuscular and synaptic functions (Miller et al., 1996; Sieburth et al., 2005). A large body of work has linked sensitivity to aldicarb, which results in paralysis and shrinkage, to hypercontraction of body wall muscles. To understand the contribution of the body wall muscles to overall stiffness of the animal, we modulated the muscle tone using aldicarb, expecting a stiffening due to muscle hyper-contraction. However, using two different force-indentation systems, we observed that administration of aldicarb reduced stiffness. The question is therefore whether the effect of aldicarb in addition to muscle contraction could also affect other properties of *C. elegans* and, if so, what would these be?

Aldicarb-treated animals shrink, and they are softer than control animals (BDM-treated). In addition, our biomechanical model suggests the most substantial change in stiffness arises from the pseudocoelom compartment. Taking these points together, we propose that aldicarb-treatment ultimately decreases the internal pressure. This reduction might be a secondary effect following an initial muscle contraction, possibly to counteract the pressure generated during muscle contraction. Animals exposed to high salt and aldicarb solution decreased in stiffness and volume, however differential modification of the width and length of the nematodes induced by these agents suggests different underlying mechanisms.

We did not observe any evidence for muscle hyper-contraction. Firstly, in our optogenetic experiments, light-activation caused a stiffening for some of the aldicarb-treated animals, suggesting their muscles were in a responsive state, as opposed to hyper-contracted. Secondly, aldicarb-treated animals are softer compared to control BDM-treated animals. Thirdly, when administering aldicarb to untreated animals in our μFDS system we observed a gradual softening of the animal (Supplementary Figure S5). Aldicarb is expected to inhibit ACE, acetyl-choline-esterase, leading to the accumulation of the neurotransmitter ACh. The main excitatory receptor in the body wall muscle is the levamisole-sensitive ACh receptor (L-AChR). In their original work, Lewis J. A. et al. (1980) described that wild-type animals exposed to levamisole rapidly contract and within a few minutes fully relax the muscle resulting in flaccid paralysis. Moreover, a recent study from the Bessereau Lab showed that exposure to levamisole beyond 10 min leads to depolarization of the muscle cells, inactivation of voltage gated calcium channels, decrease in calcium index, and ultimately muscle relaxation (Jospin et al., 2022). An accumulation of ACh at the neuro-muscular junction caused by aldicarb could similarly activate levamisole-sensitive ACh receptors and inactivate voltage gated calcium channels resulting in muscle relaxation. This may explain why we did not observe stiffening of aldicarb-treated animals. Optogenetically-induced muscle contraction requires functional voltage-gated Ca^2+^-channels (VGCC) (Nagel et al., 2005). If activation of L-AChR leads to inactivation VGCCs over time, it explains why we observed a partial response in aldicarb-treated animals in our optogenetic experiments.

A previous study by Petzold et al. (2011) measured stiffening of *C. elegans* upon muscle contraction using a set of optogenetic experiments and drug treatments including levamisole. Similar to our study, light-induced muscle contraction increased stiffness. Although the focus of our work was on the effect of aldicarb on stiffness, we also used levamisole (tetramisole) as complementary approach to assay muscle stiffness. In our experimental set-up, we did not measure stiffening of the animals upon levamisole treatment, with neither of our two force displacement methods. However, levamisole in the experimental set-up of Petzold and others, they increased the stiffness of the animal. Possible reasons for this could be the age of the animals, drug exposure and concentration. The latter two are likely influenced by the distinct animal preparation method. In our approach, we immersed freely moving animals in the drug solution until they were paralysed, and subsequently glued them for F-D measurements. In contrast, (Petzold et al., 2011), fixed unparalysed animals by gluing the head and tail onto drug infused agarose pads. Covering head and tail with glue may modify drug uptake and tissue availability to the drug, and hence affect concentration and duration of the action of the drug.

But why did the worms become softer when treated with aldicarb? There are many more ACh receptors other than those in the body wall muscles. A study by the Hobert lab (Pereira et al., 2015) describes that more than 50% of neurons in *C. elegans* (159 out of 302) are cholinergic including motor neurons, sensory neurons, and interneurons that release ACh. *Caenorhabditis elegans* expresses a large number of ACh receptors including ion-gated excitatory nicotinic AChR, muscarinic AChR and possibly inhibitory anion-gated ion channels (Treinin and Jin, 2021), which can be found on various tissues. For example, ion-gated channels can be found on pharyngeal muscles, body wall muscles, cholinergic and GABAerig motor neurons, and mechanosensory neurons. In theory, an accumulation of ACh due to the ACE inhibitor aldicarb could stimulate any ACh receptor. It is worth noting, that in a first screen for aldicarb resistance by Miller et al. (1996) only a few mutations were actually directly linked to cholinergic transmission. We suggest, that aldicarb affects other tissues apart from the body wall muscles, and that an accumulation of the neurotransmitter ACh also leads to a decrease in hydrostatic pressure.

When comparing the effects of aldicarb administered on the plate and in solution, animals on plates underwent additional shrinkage, resulting in reduced length and volume (Supplementary Figure S3A). This might be attributable to the behaviour of animals, which swim in solution and crawl on plates. Swimming animals showing diminished pharyngeal pumping in comparison to their counterparts that crawl on agar plates (Vidal-Gadea and Pierce-Shimomura, 2012). When administered on an NGM plate aldicarb is likely to undergo both digestion and absorption, enabling it to reach more tissue. Whereas animals in solution will experience reduced intestinal uptake. Although not significant, animals on plates tended to be softer than animals treated in aldicarb solution. The relationship between volume and stiffness is not necessarily linear, a reduction in volume does not have to equate to a proportional decrease in stiffness, and that there must exist a limit to how soft an animal can become due to the reduced pressure in the pseudocoelom.

BDM is a well-characterized inhibitor of skeletal muscle myosin-II (Higuchi and Takemori, 1989; Soeno et al., 1999). It has also been reported to affect many non-myosin proteins including connexins, potassium channels and L-type calcium channels (Ostap, 2002). It is used to paralyse *C. elegans* but the exact target is unknown. When Backholm et al. (2013) used it in parallel to another known nematode anaesthetic NaN_3_, they reported no difference between NaN_3_ or BDM in the bending properties of animals, stating that the muscles were relaxed. In our experiments, BDM-treated worms responded to optogenetically-induced muscle contraction with increasing stiffness in both our force-sensing systems. Hence BDM-treated animals have relaxed or responsive muscles. However, it also suggests that the paralysing effect of BDM when concentration is 148 mM does not inhibit muscle myosin in *C. elegans*, or at least not within the time frame of our experiments.

## 5 Conclusion

We investigated the biomechanics of *C. elegans* using empirical data together with an *in silico* model, using the FE method, to characterize the mechanical properties of three different tissue compartments. Building our model in a multi-compartmental way allowed us to assign individual Young’s moduli for these three compartments, i.e., cuticle and hypodermis, muscle, and pseudocoelom, based on the shape of the force-indentation curve. Using our *in silico* model, we investigated the impact of osmotic shock and the neuromuscular agents (i.e., aldicarb, tetramisol) on the mechanical properties of each tissue compartment. Despite the expected hyper-contracting muscle action of aldicarb, treatment led to a softening of the animal accompanied by shrinkage, with the largest impact observed on the pseudocoelom compartment. Optogenetically-controlled muscle contraction stiffened the animal, even under aldicarb treatment altogether, thus indicating additional effects of aldicarb. While we demonstrated the capacity of our *in silico* model to probe the impact of drugs in muscle function and hydrostatic pressure, the model can be in the future applied to investigate the biomechanical impact of mutations that, for example, affect the cuticle or muscle function, or of pharmacologicals directed to improve muscle function, or collagen production. Furthermore, the model can be used to study the impact of ageing and diet on specific tissues mechanics.

## Data Availability

The raw (experimental and simulation) data supporting the findings of this study are publicly available. This data can be found here: https://doi.org/10.6084/m9.figshare.25303960 and https://doi.org/10.6084/m9.figshare.25304035.
