# Supplementary figures and images for: The influence of internal pressure and neuromuscular agents on C. elegans biomechanics: an empirical and multi-compartmental in silico modelling study
Source: Front Bioeng Biotechnol. 2024 Mar 15;12:1335788. doi: 10.3389/fbioe.2024.1335788 (PMC10978802; doi:10.3389/fbioe.2024.1335788)

# Supplementary Figure 5

a

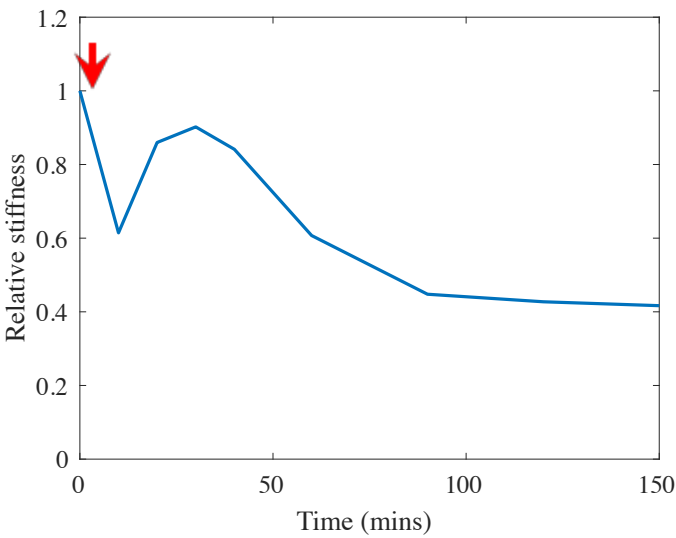

b

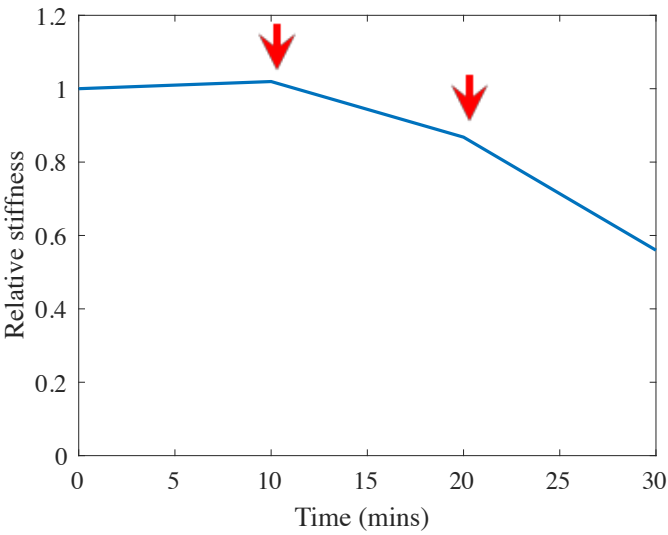

Supplement: Supplementary file 1 [file Image5.pdf]

# Supplementary Figure 4

a

Micro-Force-Displacement System ( $\mu$ FDS)

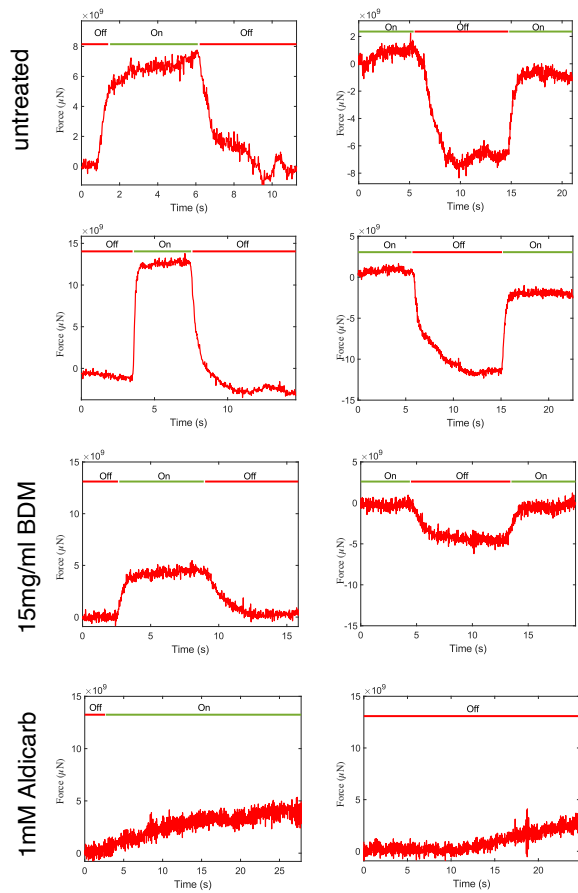

b

atomic force microscope

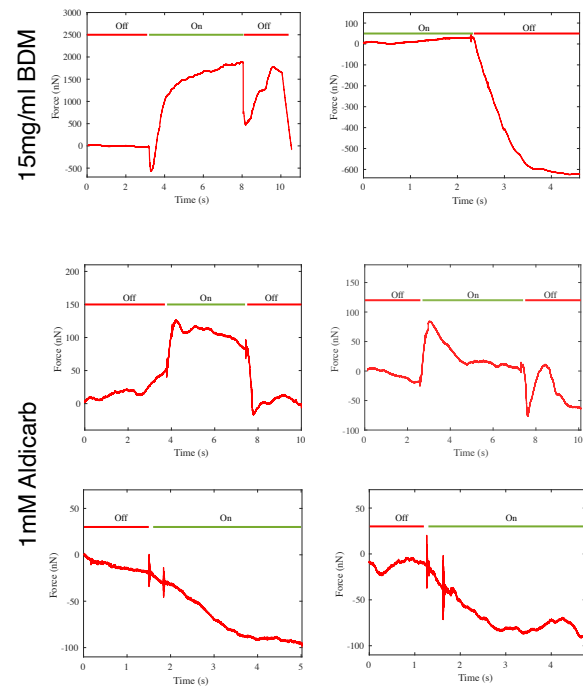

Supplement: Supplementary file 2 [file Image4.pdf]

# Supplementary Figure 2

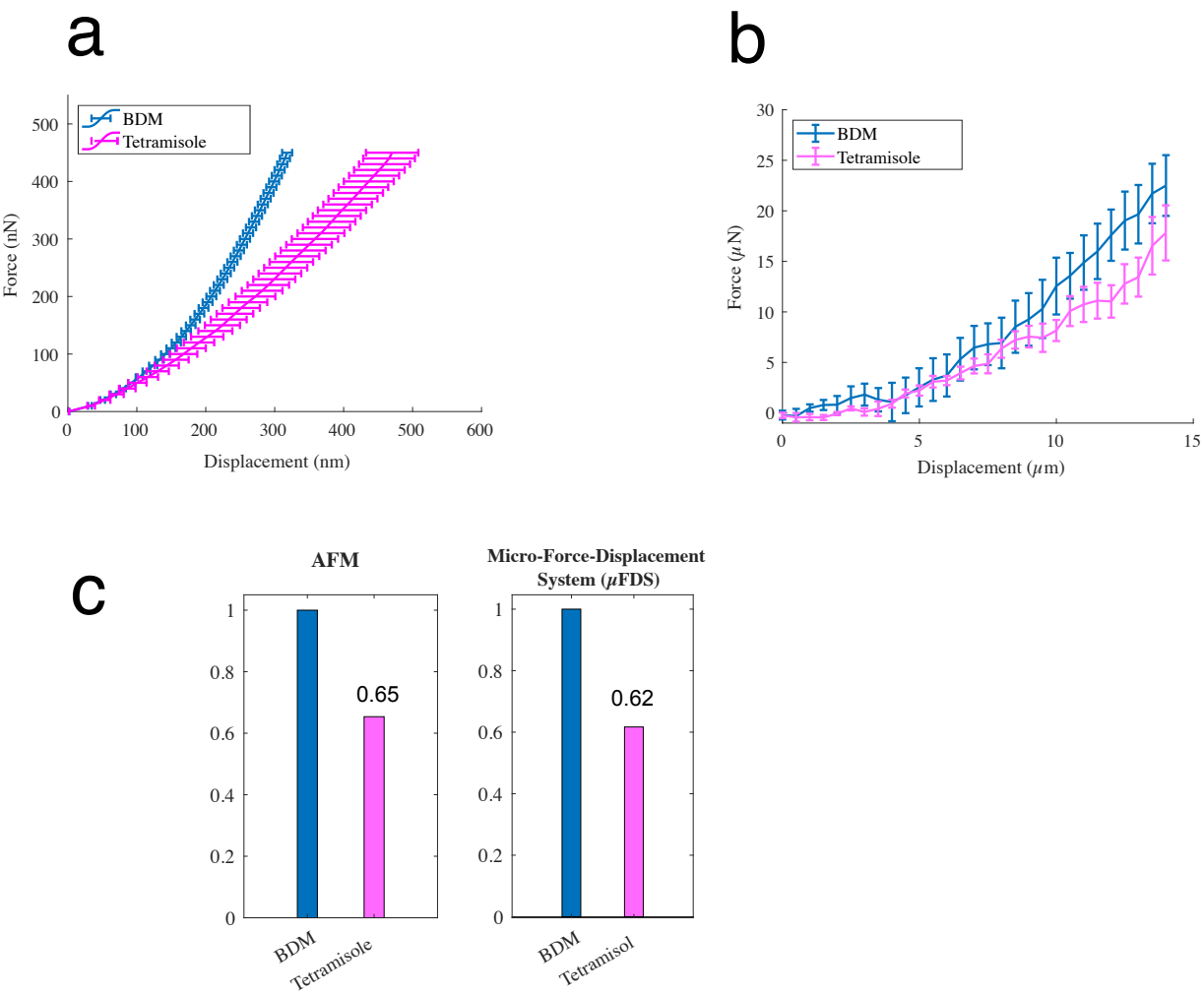

Supplement: Supplementary file 3 [file Image2.pdf]

# Supplementary Figure 3

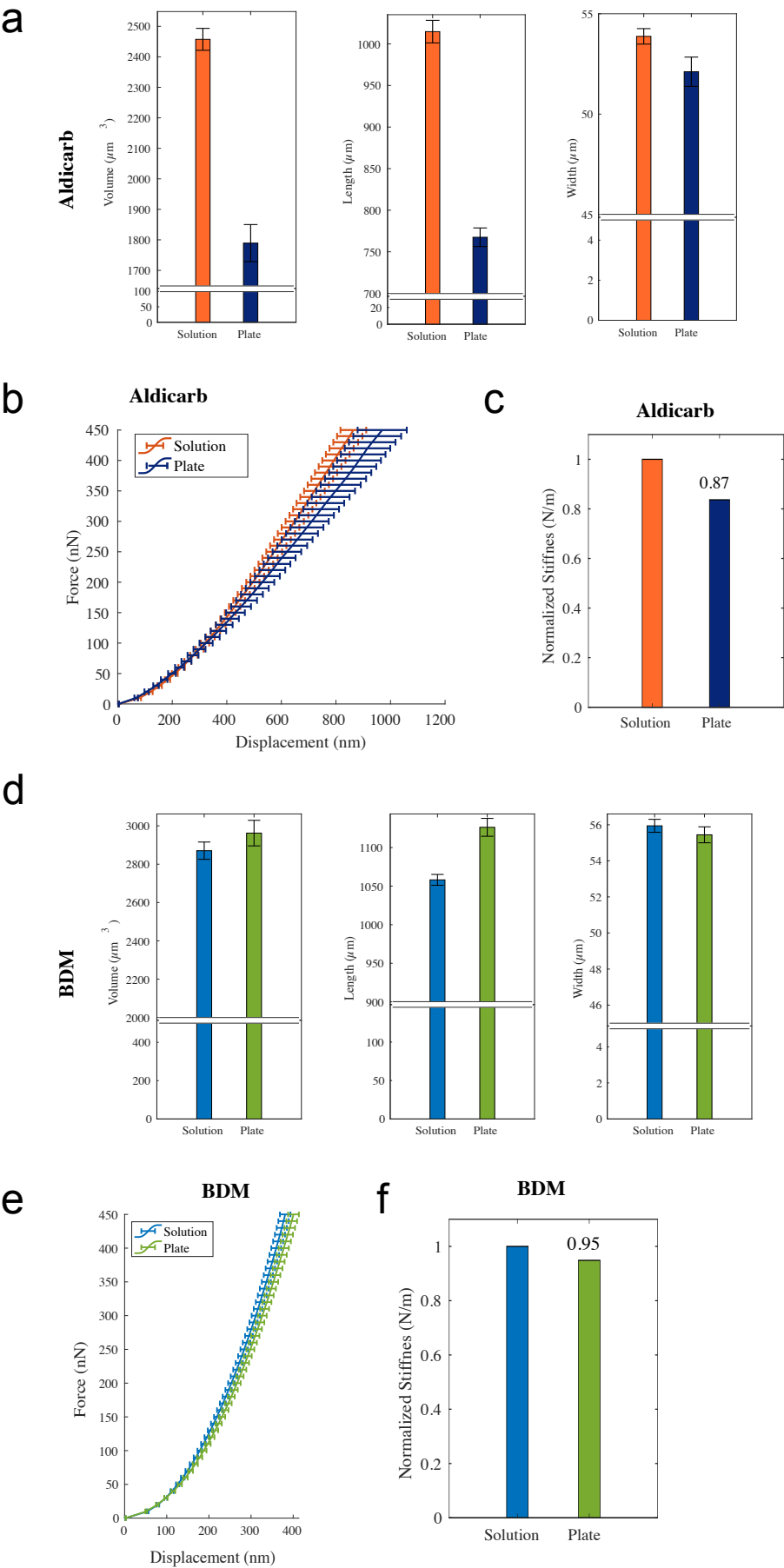

Supplement: Supplementary file 4 [file Image3.pdf]

# Supplementary Figure 1

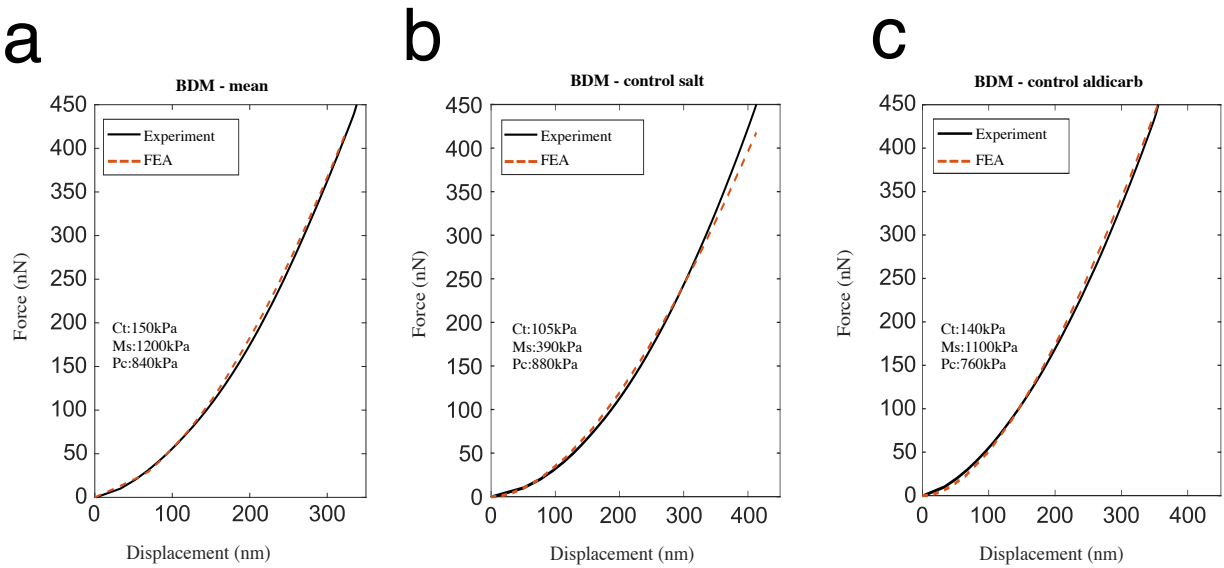

Supplement: Supplementary file 7 [file Image1.pdf]
