# Supplementary material for: The influence of internal pressure and neuromuscular agents on C. elegans biomechanics: an empirical and multi-compartmental in silico modelling study
Source: Front Bioeng Biotechnol. 2024 Mar 15;12:1335788. doi: 10.3389/fbioe.2024.1335788 (PMC10978802; doi:10.3389/fbioe.2024.1335788)
Supplement: Supplementary file 5 [file DataSheet1.PDF]

# Supplementary Table 1

## The influence of internal pressure and neuromuscular agents on *C. elegans* biomechanics: an empirical and multi-compartmental *in silico* modelling study

C.L. Essmann, M. Elmi, C. Rekatsinas, N. Chrysochoidis, M. Shaw, V. Pawar, M.A. Srinivasan, V. Vavourakis

Bulk stiffness of the *Caenorhabditis elegans* (*C. elegans*) used to produce the normalized bar plots, see first column of the table, in the manuscript.

| | **BDM** | **Salt** |
|---|---|---|
| **Fig 2b** | 1.64 N/m | 0.492 N/m |
| | **BDM** | **Aldicarb** |
| **Fig 3d** | 2 N/m (*left*); 1.46 N/m (*right*) | 0.698 N/m (*left*); 0.453 N/m (*right*) |
| | **BDM** | **Tetramisol** |
| **Suppl Fig 2c** | 2.2 N/m (*left*); 2 N/m (*right*) | 1.43 N/m (*left*); 1.24 N/m (*right*) |
| | **Aldicarb** Solution | **Aldicarb** Plate |
| **Suppl Fig 3c** | 0.698 N/m | 0.607 N/m |
| | **BDM** Solution | **BDM** Plate |
| **Suppl Fig 3f** | 2.24 N/m | 2.128 N/m |
